# Supplementary material for: Repeat-Specific Functions for the C-Terminal Domain of RNA Polymerase II in Budding Yeast
Source: G3 (Bethesda). 2018 Mar 9;8(5):1593–601. doi: 10.1534/g3.118.200086 (PMC5940151; doi:10.1534/g3.118.200086)
Supplement: Supplementary file 1 [file 1593FileS1.pdf]

Table S1: Plasmids used in this work

Name	Reference	Description
pJMD2	McDaniel et al. 2010	Vector used for CTD plasmid construction by recursive directional ligation.
pRS315	Sikorski RS and Hieter P 1989	Empty LEU2 vector. Refered to as pLEU2 in the text.
pRPB1	Morill et al. 2016	Plasmid with copy of RPB1 containing 26 wildtype CTD repeats, refered to as "WT" in the text.
pRPB1-CTD$_{8}$	Morill et al. 2016	pRPB1 with 8 consensus CTD repeats.
pRPB1-CTD$_{10}$	Morill et al. 2016	pRPB1 with 10 consensus CTD repeats.
pRPB1-CTD$_{14}$	Morill et al. 2016	pRPB1 with 14 consensus CTD repeats.
pRPB1-CTD$_{26}$	Morill et al. 2016	pRPB1 with 26 consensus CTD repeats.
pCTD$_{26}$-S>A$_{2-9}$	This work	pRPB1 with all S to A mutations in only repeats 2-9.
pCTD$_{26}$-S>A$_{10-17}$	This work	pRPB1 with all S to A mutations in only repeats 10-17.
pCTD$_{26}$-S>A$_{18-25}$	This work	pRPB1 with all S to A mutations in only repeats 18-25.
pCTD$_{26}$-S2A$_{2-9}$	This work	pRPB1 with only S2 to A2 mutations in repeats 2-9.
pCTD$_{26}$-S5A$_{2-9}$	This work	pRPB1 with only S5 to A5 mutations in repeats 2-9.
pCTD$_{26}$-S7A$_{2-9}$	This work	pRPB1 with only S7 to A7 mutations in repeats 2-9.
pCTD$_{26}$-S>A$_{2-9\ \Delta 4}$	This work	Suppressor mutation of pCTD$_{26}$-S>A$_{2-9}$ where 4 repeats have been deleted.
pCTD$_{26}$-S>A$_{2-9\ \Delta 6}$	This work	Suppressor mutation of pCTD$_{26}$-S>A$_{2-9}$ where 6 repeats have been deleted.
pCTD$_{26}$-S>A$_{10-17\ \Delta 4}$	This work	Suppressor mutation of pCTD$_{26}$-S>A$_{10-17}$ where 4 repeats have been deleted.
pCTD$_{26}$-S>A$_{10-17\ \Delta 6}$	This work	Suppressor mutation of pCTD$_{26}$-S>A$_{10-17}$ where 6 repeats have been deleted.
pCTD$_{26}$-S>A$_{10-17\ \Delta 12-21\ \wedge 2-17}$	This work	Suppressor mutation of pCTD$_{26}$-S>A$_{10-17}$ where repeats 12 to 21 have been deleted and repeats 2 to 17 have been duplicated and inserted.

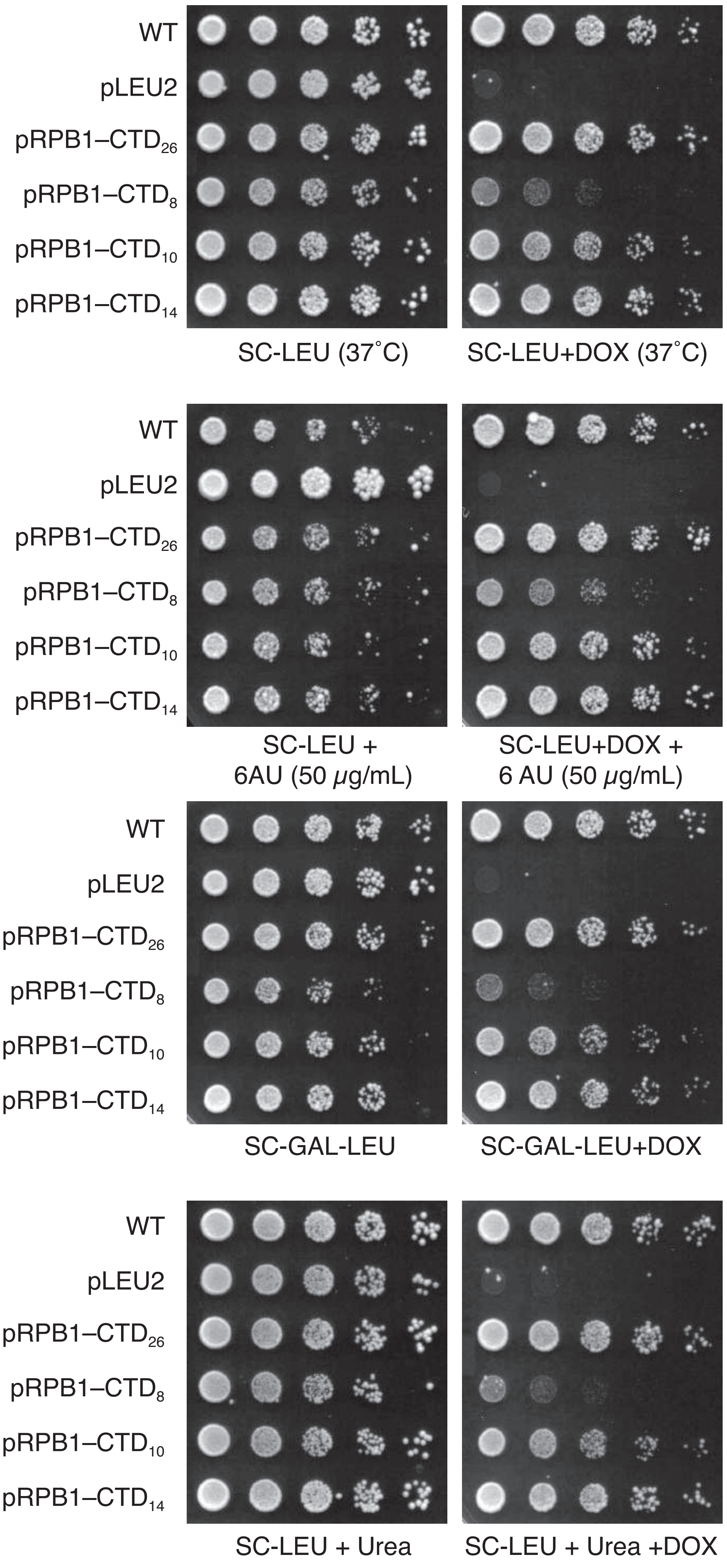

WT
pLEU2
pRPB1–CTD26
pRPB1–CTD8
pRPB1–CTD10
pRPB1–CTD14
SC-LEU (37˚C)
SC-LEU+DOX (37˚C)
WT
pLEU2
pRPB1–CTD26
pRPB1–CTD8
pRPB1–CTD10
pRPB1–CTD14
SC-LEU + 6AU (50 μg/mL)
SC-LEU+DOX + 6 AU (50 μg/mL)
WT
pLEU2
pRPB1–CTD26
pRPB1–CTD8
pRPB1–CTD10
pRPB1–CTD14
SC-GAL-LEU
SC-GAL-LEU+DOX
WT
pLEU2
pRPB1–CTD26
pRPB1–CTD8
pRPB1–CTD10
pRPB1–CTD14
SC-LEU + Urea
SC-LEU + Urea +DOX

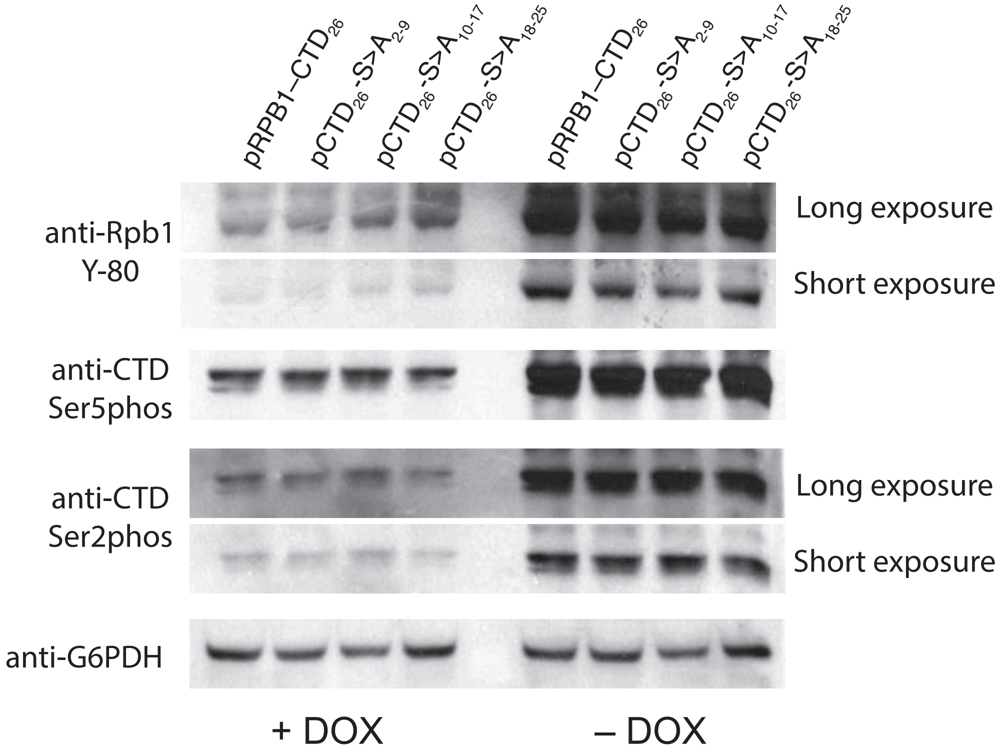
pRPB1–CTD26
pCTD26-S>A2-9
pCTD26-S>A10-17
pCTD26-S>A18-25
pRPB1–CTD26
pCTD26-S>A2-9
pCTD26-S>A10-17
pCTD26-S>A18-25
anti-Rpb1
Y-80
Long exposure
Short exposure
anti-CTD
Ser5phos
anti-CTD
Ser2phos
Long exposure
Short exposure
anti-G6PDH
+ DOX
– DOX

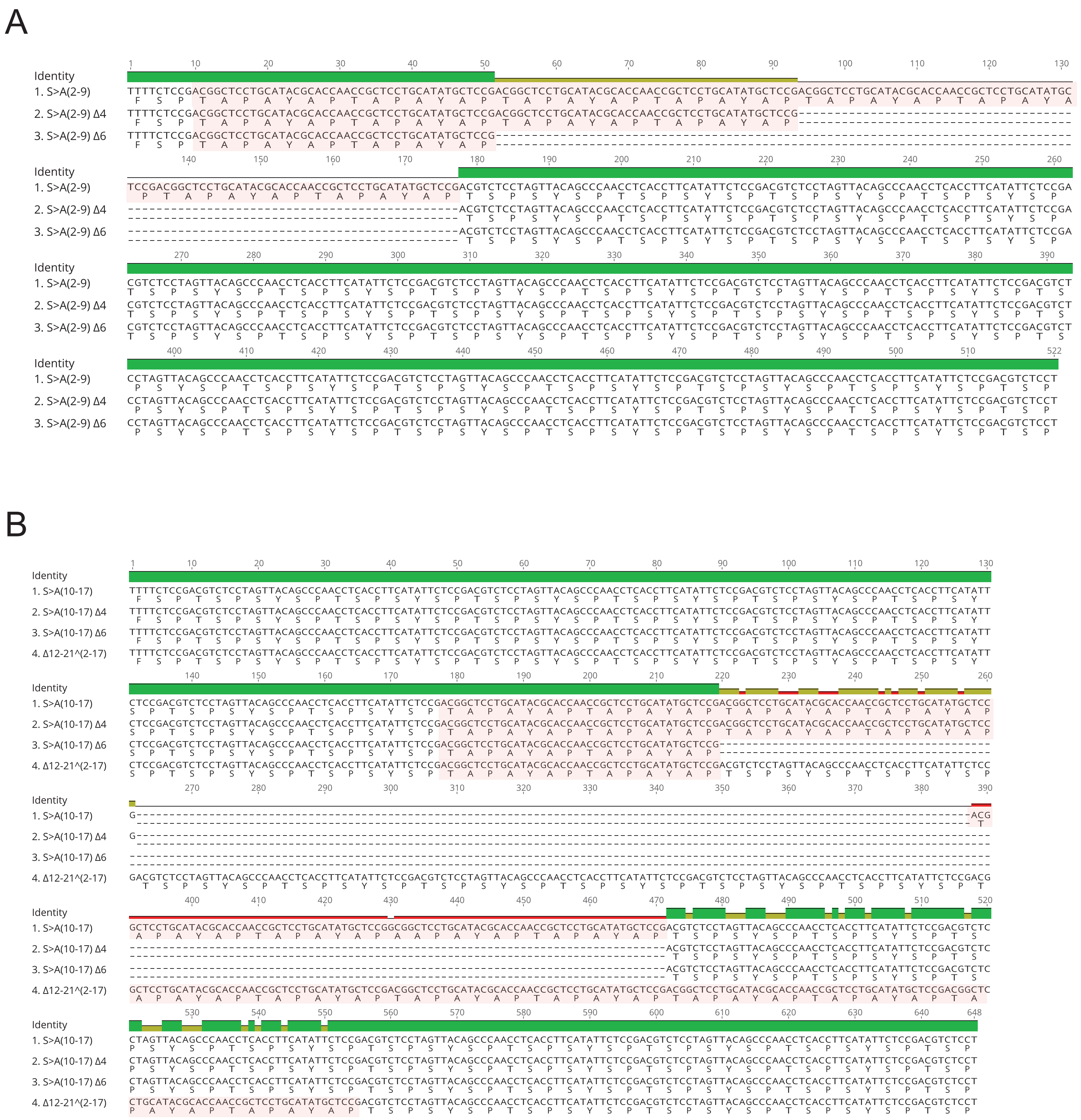
A
Identity
1. S>A(2-9)
2. S>A(2-9) Δ4
3. S>A(2-9) Δ6
B
Identity
1. S>A(10-17)
2. S>A(10-17) Δ4
3. S>A(10-17) Δ6
4. Δ12-21^(2-17)

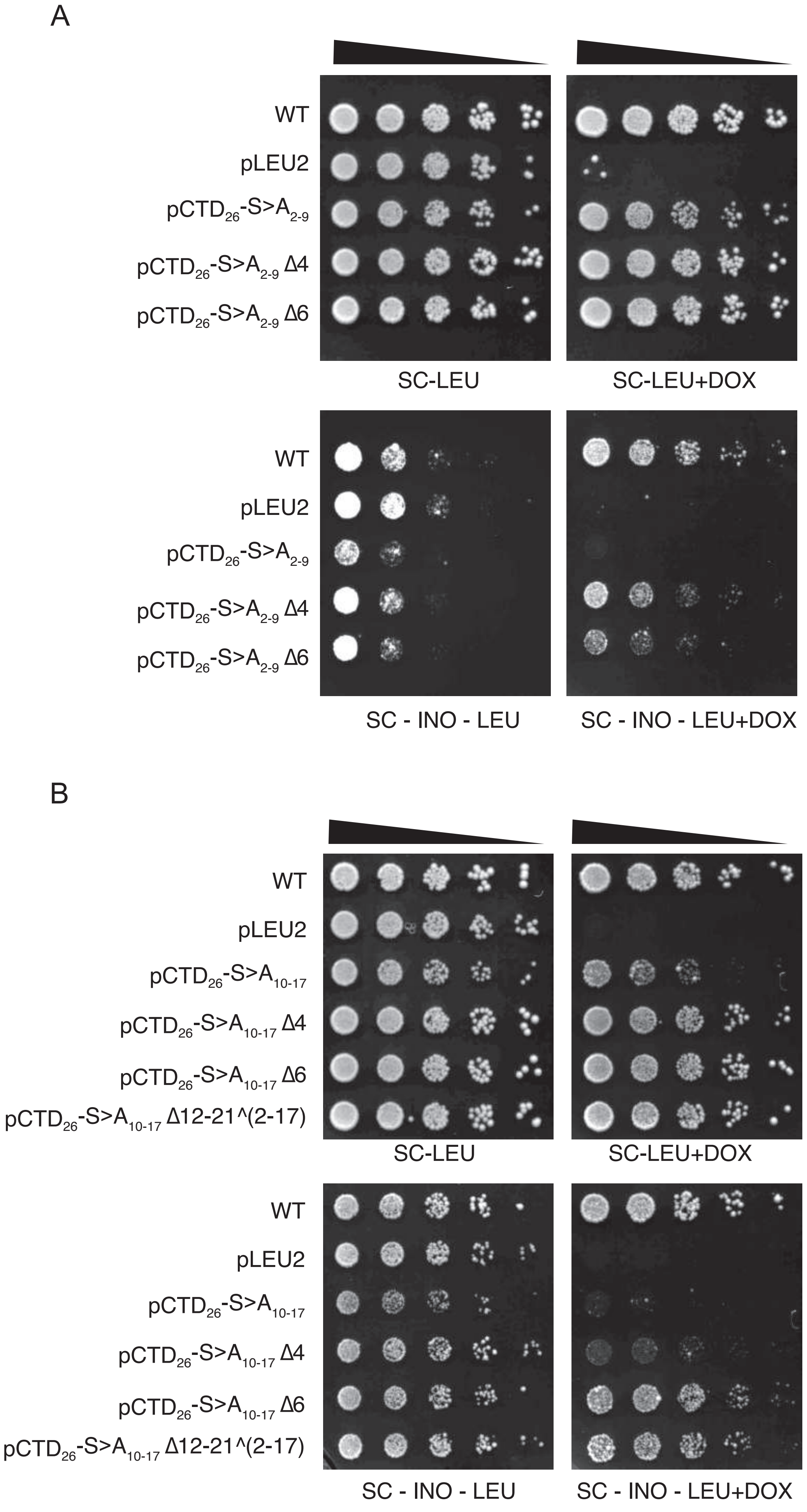
A
WT
pLEU2
pCTD26-S>A2-9
pCTD26-S>A2-9 Δ4
pCTD26-S>A2-9 Δ6
SC-LEU
SC-LEU+DOX
SC - INO - LEU
SC - INO - LEU+DOX
B
WT
pLEU2
pCTD26-S>A10-17
pCTD26-S>A10-17 Δ4
pCTD26-S>A10-17 Δ6
pCTD26-S>A10-17 Δ12-21^(2-17)
SC-LEU
SC-LEU+DOX
SC - INO - LEU
SC - INO - LEU+DOX

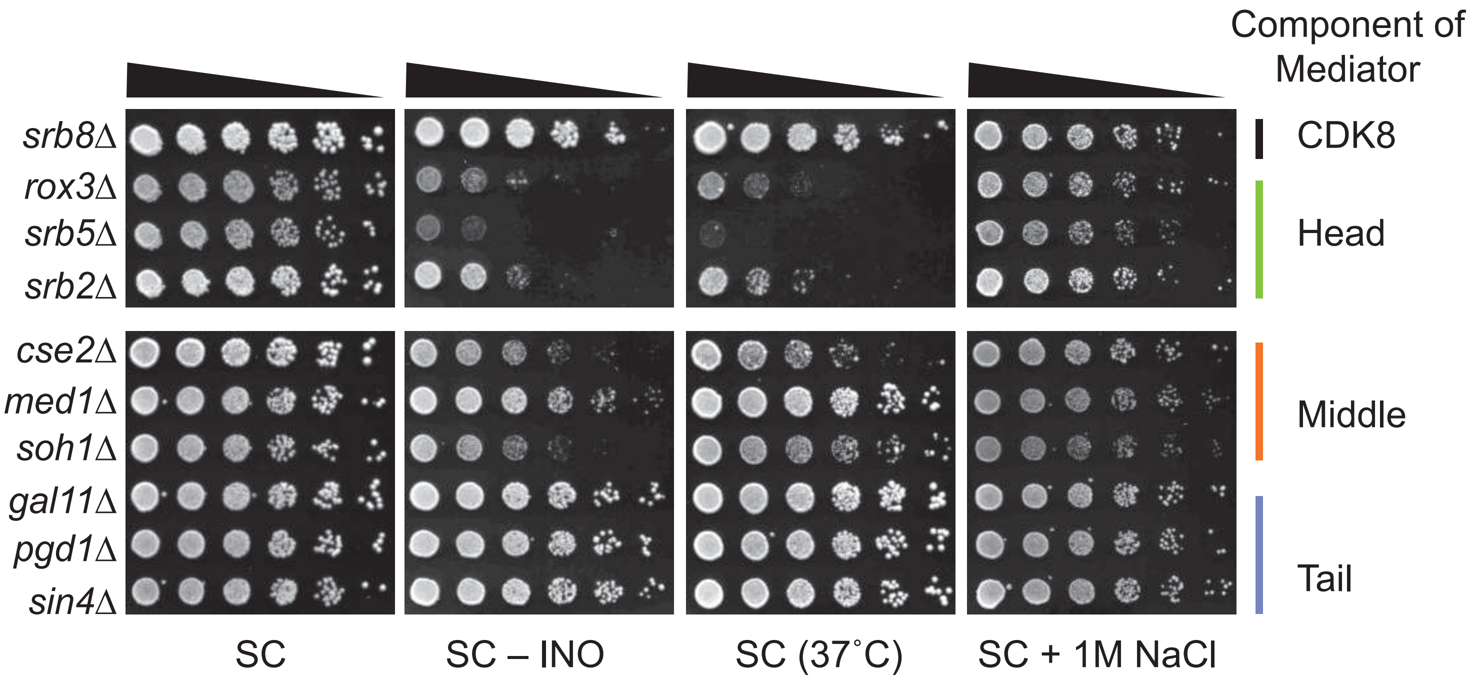
Component of Mediator
srb8Δ
rox3Δ
srb5Δ
srb2Δ
cse2Δ
med1Δ
soh1Δ
gal11Δ
pgd1Δ
sin4Δ
CDK8
Head
Middle
Tail
SC
SC – INO
SC (37˚C)
SC + 1M NaCl
